# Diversity of *Mycoplasma hominis* clinical isolates from Bordeaux, France, as assessed by multiple-locus variable-number tandem repeat analysis

**DOI:** 10.1186/1471-2180-13-120

**Published:** 2013-05-28

**Authors:** Cyril Férandon, Olivia Peuchant, Hélène Renaudin, Cécile Bébéar

**Affiliations:** 1 Univ. Bordeaux, USC Mycoplasmal and Chlamydial Infections in Humans, 33076, Bordeaux, France; 2 INRA, USC Mycoplasmal and Chlamydial Infections in Humans, 33076, Bordeaux, France; 3 CHU de Bordeaux, Laboratoire de Bactériologie, 33076, Bordeaux, France

**Keywords:** *Mycoplasma hominis*, MLVA, Genotyping

## Abstract

**Background:**

*Mycoplasma hominis* is an opportunistic human mycoplasma species that can cause various urogenital infections and, less frequently, extragenital infections. The objective of this work was to study the genetic diversity of this species using a molecular typing method based on multiple-locus variable-number tandem repeat (VNTR) analysis (MLVA).

**Results:**

The genome content of *M. hominis* PG21 was analysed for tandem repeats (TRs), and five of the 130 TRs identified were selected for use in an MLVA assay. The method was based on GeneScan analysis of VNTR loci using multiplex PCR with fluorescent dyes and resolution by capillary electrophoresis. This approach was used on a collection of 210 urogenital and extragenital French clinical isolates collected between 1987 and 2009. Forty MLVA types were found. The discriminatory index of our MLVA scheme was 0.924. Using this new typing tool, persistent infection was suggested for six patients and new infection for one patient. Furthermore, mother-to-child transmission was confirmed in the two cases studied. Application of MLVA to a wide range of *M. hominis* isolates revealed high genotypic diversity and no obvious link between the MLVA type and the isolate year of collection, the patient’s age or sex, the anatomical origin of the isolates or resistance to antibiotics was found.

**Conclusions:**

Our MLVA scheme highlights the high genetic heterogeneity of the *M. hominis* species. It seems too discriminatory to be used for large epidemiological studies but has proven its usefulness for molecular studies at the individual level.

## Background

*Mycoplasma hominis* is an opportunistic human mycoplasma species that resides in the lower urogenital tract as a commensal pathogen. This species has been implicated in bacterial vaginosis (BV), pelvic inflammatory disease, infection during pregnancy, preterm labour and neonatal infections [1]. The occurrence of *M. hominis* organisms in a large number in the vagina and cervix is recognized as being associated strongly with BV. *M. hominis* organisms and other BV-associated bacteria in the vaginal and cervical specimens, quite frequently invaded the endometrium sometimes with an antibody response [2,3]. *M. hominis* has been isolated from the endometria and fallopian tubes of about 10% of women with salpingitis at laparoscopy and accompanied by specific antibodies [4]. More recently, Taylor-Robinson *et al.* reported that of 22 women with salpingitis at laparoscopy, *M. hominis* was a questionable sole cause in one woman and the primary or equal primary contributor in three [3]. In this study, *M. hominis* in a large number (≥ 10^4^-10^5^ color changing units -CCU- /ml) in the vagina and cervix were detected most often in women with salpingitis at laparoscopy. However, the significance of this mycoplasma, especially when associated with BV, can be difficult to assess when several microorganisms are present [3,5]. Otherwise*, M. hominis* has been linked to a variety of extragenital infections, such as septicaemia, septic arthritis, wound infection, brain and perirenal abscesses, mediastinistis and other infections in immunocompromised patients [5]. Any assessment of the pathogenic potential of *M. hominis* is complicated by the high degree of genomic and antigenic heterogeneity observed within the species.

A few molecular typing methods have been developed for *M. hominis*. Pulse-field gel electrophoresis (PFGE) [6,7], restriction fragment length polymorphism (RFLP) analysis [8], amplified fragment length polymorphism (AFLP) [9] and random amplified polymorphic DNA (RADP) [10] have been used to study the genetic diversity of this species. However, these methods are time-consuming, require a relatively large amount of biological material, may be difficult to reproduce and standardise between laboratories and generate results that are difficult to interpret.

Other molecular typing methods based on sequence analyses of the *p75*, *p120’* and *vaa* genes have been developed [11-13]. An MLST approach based on the sequence analysis of six housekeeping genes and one gene encoding a membrane protein was conducted for 20 *M. hominis* isolates [14]. However, this method was used to estimate the frequency of recombination in *M. hominis*, rather than for genotyping.

Multiple locus variable-number tandem-repeat (VNTR) analysis (MLVA) is new genotyping method based on the variation in the copy numbers of tandem repeat (TR) sequences at different genomic loci among isolates. MLVA has been used successfully to subtype certain *Mycoplasma* species [15-19].

Using the recently described *M. hominis* PG21 genome sequence [20], we developed an automated MLVA scheme, without a sequencing step, for *M. hominis* typing. This method was subsequently applied to a wide range of *M. hominis* clinical isolates from genital and extragenital infections collected between 1987 and 2009. We used MLVA to assess *M. hominis* genotypic diversity and characterise the pattern of human infections.

## Methods

### Ethics statement

The present project is in compliance with the Helsinki Declaration (Ethical Principles for Medical Research Involving Human Subjects).

The study was conducted in accordance with the guidelines of the “Direction de la Recherche Clinique et de l’Innovation”, the research board of Bordeaux University hospital, Bordeaux, France. All patient data were anonymously reported, with no possibility of connecting the isolates and specimens to individual patients. Using the written “livret d’accueil” of the Bordeaux University Hospital, patients are explicitly informed at the admission to hospital that their samples could be used for research purposes and that they can oppose to this use. As specimens used in this study are part of routine patient management without any additional sampling, and since patients provided no objection for their samples to be used, the article L1211-2 of the French code of Public Health states that this study did not need to be examined by the ethical committee “Comité de Protection des Personnes” and that patient’s informed consent was not required.

### Bacterial strains, culture and DNA preparation

The PG21 (ATCC 23114), M132 (ATCC 43521) and H34 (ATCC 15056) *M. hominis* reference strains and 207 French clinical isolates collected between 1987 and 2009 were used in this study (Additional file 1: Table S1). The 167 urogenital clinical isolates were collected at the Bordeaux University Hospital and obtained from i) specimens where *M. hominis* was present as a commensal, i.e. cervical samples with titres of *M. hominis* < 10^4^ CCU /ml and male specimens, ii) cervical swabs from patients with titres of *M. hominis* ≥ 10^4^ CCU /ml without association with BV, iii) cervical swabs from female patients with titres of *M. hominis* ≥ 10^4^ CCU /ml and suffering from bacterial vaginosis, iv) vaginal swabs from pregnant women with threatened preterm delivery whatever the titre of *M. hominis*, v) specimens from women presenting upper genital tract infection whatever the titre of *M. hominis*, these specimens being normally sterile. Thirty-four isolates obtained from extragenital specimens and collected at hospitals from 10 different French cities were also tested. Finally, we genotyped six isolates obtained from two mother-neonate pairs.

Among these 210 isolates, concomitant and sequential isolates were obtained for one and seven patients, respectively.

Antibiotic susceptibility testing, realised when *M. hominis* was in a pathogenic situation, showed that 66 urogenital isolates were resistant to tetracyclines, seven extragenital isolates were resistant to ofloxacin, two urogenital isolates were resistant to both tetracyclines and ofloxacin and 91 isolates presented a wild-type profile.

The growth conditions used for the *M. hominis* isolates have been described previously [21]. The DNA was extracted using the MagNA Pure LC DNA isolation kit I (Roche, Meylan, France) according to the manufacturer’s instructions.

### MLVA analysis

Tandem repeat (TR) sequences were identified in the *M. hominis* PG21 genome [20] using the Tandem Repeats Finder programme (http://tandem.bu.edu/trf/trf.html) [22]. Loci were chosen if they had >80% matches between the DNA sequences of the repeat units. A total of 130 TRs were selected and designated by the letters Mho followed by a number corresponding to the order in which the TR was detected. To screen for variability in the number of TRs, PCR primers targeting the regions flanking TR loci were designed and tested on a set of 12 *M. hominis* isolates, including the PG21, M132 and H34 references strains, three urogenital isolates, one from amniotic fluid, one isolate from peritoneal fluid, one from joint fluid, one from bronchial aspirate, one from abdominal wall abscess and one from a renal abscess, collected between 1995 to 2008 from different geographical areas (Additional file 1: Table S1). Each locus was amplified individually and analysed by conventional agarose gel electrophoresis.

To confirm that length polymorphisms were the result of repeat copy number variations, the PCR products were purified using the Wizard PCR Preps DNA Purification System (Promega, Charbonnières-les-Bains, France) and double-strand sequenced (Additional file 2: Figure S1). This approach showed that only seven loci were polymorphic with different allele sizes. After evaluation of a large collection of *M. hominis* isolates, two of these seven VNTRs were rejected due to a lack of adequate discrimination, and the five remaining VNTR loci were chosen for further assessment.

The five VNTR markers ultimately selected for use in MLVA were multiplexed in two solutions named T1 and T2. The markers Mho-50, Mho-52 and Mho-53 were amplified using the solution T1, and the markers Mho-114 and Mho-116 were amplified using the solution T2. The amplifications were performed with a Mastercycler ep Gradient S thermocycler (Eppendorf, Hamburg, Germany) in a final volume of 25 μl. The reaction mixtures contained 1X Qiagen PCR buffer with 1.5 mM MgCl_2_, 0.2 mM deoxynucleotide triphosphate, 3 mM MgCl_2,_ 0.625 U of Hot Start *Taq* DNA polymerase (Qiagen, Hilden, Germany), 0.125 μM of each primer and 1 μl of template DNA from clinical isolates. The forward primers were fluorescently labelled at the 5’ end using 4,4,7,2’,4’,5’,7’-hexachloro-6-carboxy-fluorescein (HEX), 6-carboxyfluorescein (FAM; Eurogentec, Angers, France) or NED (2’-chloro-5’-fluoro-7’,8’-fused phenyl-1,4-dichloro-6-carboxyfluorescein; Applied Biosystems, Life Technologies, Carlsbad, CA, USA), depending on the locus to be amplified (Additional file 3: Table S2). All of the solutions were run under the same cycling conditions: 95°C for 15 min followed by 25 cycles of 95°C for 1 min, 56°C for 1 min and 72°C for 1 min with a final extension at 72°C for 10 min. Prior to GeneScan analysis, 0.3 μl of GeneScan ROX 500 size standard (Applied Biosystems) was added to 1 μl of each PCR product. After heat denaturation for 5 min at 95°C, the fragments were separated using an ABI 3130 Genetic Analyzer (Applied Biosystems). The GeneScan data were subsequently analysed using GeneMapper software (version 3.7; Applied Biosystems) to perform sizing and to calculate the number of repeats in the PCR fragments. Each locus was identified according to colour fluorescence. An allele number string based on the number of repeats at each locus was assigned to each isolate.

### Data analysis

The calculated numbers of repeats were imported into BioNumerics (version 6.1; Applied Maths). A minimum spanning tree (MST) was generated to visualise the relationships between the large number of isolates in a single compact image. The MST was created based on the categorical coefficient and a priority rule consisting of the highest number of single-locus variants. The polymorphism index of individual or combined VNTRs was calculated using the Hunter-Gaston discriminatory index (HGDI) [23].

## Results

### Identification of VNTRs for MLVA typing

Among the 130 TRs tested, only five were polymorphic with different allele sizes, making them useful for discriminating among types. The five VNTRs selected are distributed around the genome from nucleotide positions 181200 to 298794 in the *M. hominis* PG21 reference strain (Table 1). The PCR products ranged in size from 153 to 290 bp in the *M. hominis* PG21 reference strain. All of the VNTRs were located in open reading frames (ORFs). Markers Mho-52, Mho-53 and Mho-116 were located in the *rpoD* gene encoding the RNA polymerase sigma factor RpoD, the *pgsA* gene encoding the CDP-diacylglycerol-glycerol-3-phosphate-3-phosphatidyl transferase and the *oppA* gene encoding the oligopeptide ABC transporter substrate-binding protein, respectively. The two other markers were located in ORFs encoding hypothetical proteins. The sizes of the unit repeats ranged from 3 bp to 42 bp. Sequencing the PCR products of different sizes at each of the five loci from each of the 12 screening isolates confirmed the sizes and sequences of the individual VNTR loci.

**Table 1:** Characteristics of the five VNTR markers

**Name**	**Nucleotide position**^**a **^**(bp)**	**Locus (protein no. in the genome sequence)**	**Repeat size (bp)**	**Consensus sequence**	**% identity between VNTRs**	**HGDI**^**b**^
Mho-50	298627-298794	Hypothetical protein, predicted lipoprotein (MHO_2440)	42	TCAAGATTCTACAACCACAGGTGAAGATTCGACTGGACAATC	98	0.313
Mho-52	259317-259340	*rpoD* gene (MHO_2150)	3	GAT	82	0.203
Mho-53	246308-246325	*pgsA* gene (MHO_2070)	3	ATT	100	0.784
Mho-114	190335-190346	Hypothetical protein, predicted lipoprotein (MHO_1590)	6	TTGGCT	100	0.336
Mho-116	181200-181202	*oppA* gene (MHO_1510)	3	GAA	100	0.020

^a^Position (5’ end) on the *M. hominis* PG21 genome sequence.

^b^HGDI, Hunter Gaston Diversity Index.

The stability of the five polymorphic markers in five strains was examined after 10 serial passages in Hayflick modified broth medium supplemented with arginine. The analysis of the five strains resulted in identical MLVA profiles for all markers.

The use of fluorescently labelled primers in two multiplex PCRs (Mho-50, Mho-52 and Mho-53 for PCR T1 and Mho-114 and Mho-116 for the PCR T2), and capillary electrophoresis facilitated the interpretation of the results, an improvement over the standard agarose gel electrophoresis. Using GeneMapper Software, all loci were clearly identified on electropherograms according to their size ranges and colours, and the amplicon sizes allowed the determination of repeat number.

### MLVA typing of clinical isolates

The set of five VNTRs was used to type a large collection of 210 *M. hominis* French clinical isolates. All of the VNTRs were efficiently amplified in each *M. hominis* isolate tested. The size variation of the amplicons was exact multiples of the repeats (Table 2). This was confirmed by sequencing amplicons which presented an unexpected size variation using the capillary electrophoresis analysis. The marker Mho-53 was the most discriminatory VNTR, displaying six different allele sizes with repeat copy numbers ranging from 3 to 8, depending on the isolate. The markers Mho-50 and Mho-52 showed five and three different allele sizes, respectively. The other markers yielded only two different-sized PCR products. The marker Mho-116 was the most homogenous marker, as almost all of the isolates harboured one repeat (three harboured two copies). This finding was reflected by the diversity index of each VNTR, estimated from the HGDI, with a value of 0.784 for the most discriminatory marker (Mho-53) and a value of 0.020 for the less discriminatory one (Mho-116). The overall discriminatory index of the MLVA assay was 0.924.

**Table 2:** Number of repeat units for the five VNTR markers

**MLVA type**	**No. of repeats at the following VNTR loci**				
	**Mho-50**	**Mho-52**	**Mho-53**	**Mho-114**	**Mho-116**
**1**	1	8	8	1	1
**2**	1	8	3	1	1
**3**	1	8	3	2	1
**4**	1	8	4	1	1
**5**	1	8	4	2	1
**6**	1	8	4	2	2
**7**	1	8	5	1	1
**8**	1	8	5	2	1
**9**	1	8	6	1	1
**10**	1	8	6	2	1
**11**	1	8	7	1	1
**12**	1	8	7	2	1
**13**	1	8	8	2	1
**14**	3	8	3	1	1
**15**	1	9	3	2	1
**16**	1	9	4	1	1
**17**	1	9	4	2	1
**18**	1	9	5	2	1
**19**	2	8	3	1	1
**20**	2	8	3	2	1
**21**	2	8	4	1	1
**22**	2	8	4	2	1
**23**	2	8	5	2	1
**24**	2	9	7	1	1
**25**	3	8	3	2	1
**26**	3	8	4	1	1
**27**	3	8	4	2	1
**28**	3	8	5	2	1
**29**	3	8	6	2	1
**30**	3	8	7	2	1
**31**	3	9	4	2	1
**32**	3	9	7	2	1
**33**	4	8	3	2	2
**34**	4	8	4	2	1
**35**	4	8	5	2	1
**36**	4	8	6	2	1
**37**	5	8	4	2	1
**38**	1	10	3	2	1
**39**	1	10	4	2	1
**40**	1	10	5	2	1

A combined analysis of the five VNTR loci in the 210 *M. hominis* isolates revealed 40 MLVA types (Table 2). Three MLVA types, 5, 8 and 10, were present in more than 20 isolates. In 18 cases, one unique MLVA type was observed in a single patient.

Interestingly, the two ATCC strains, H34 and M132, had the identical MLVA type 10, while the PG21 ATCC strain belonged to the MLVA type 36. The 167 urogenital isolates were classified into 34 MLVA types (Additional file 1: Table S1). The 34 extragenital isolates contained 14 MLVA types, including eight MLVA types that had already been described for urogenital isolates.

One set of two concomitant extragenital isolates (Mh-2537, Mh-2539) collected from the same patient was analysed. The MLVA typing led to an identical MLVA profile for both isolates (Additional file 1: Table S1). Sequential isolates (obtained on time intervals from one week to six months) from each of seven patients revealed no intra-individual variation in six of them, suggesting that they suffered from a persistent *M. hominis* infection. For the seventh patient, the first isolate (Mh-4642) from the cervix was of MLVA type 2, while the second cervical isolate (Mh-4724), obtained three months later, belonged to the MLVA type 8, which suggests a novel *M. hominis* infection.

Isolates collected from two mother-neonate pairs were also analysed (Additional file 1: Table S1). In the first case, isolates obtained from the bloodstream of the mother and her new born infant belonged to the same MLVA type 8. In the second case, isolates obtained from the blood culture and cervix of the mother yielded the MLVA type 7, as well as isolates collected from blood culture and throat sample of her neonate.

**Figure 1:**
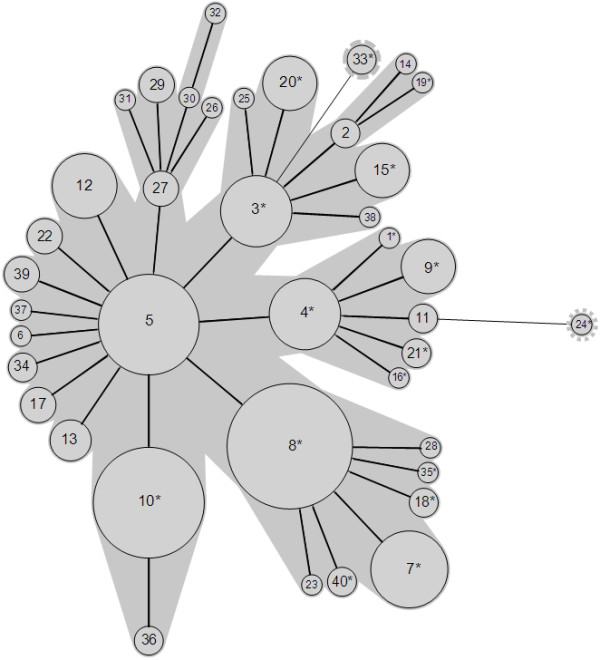
**Minimum spanning tree of 210** ***M. hominis *isolates based on categorical analysis of five VNTRs.** Each circle represents a unique MLVA profile, as indicated by a number. The size of each circle is proportional to the number of isolates belonging to the indicated MLVA type. Thick connecting lines represent one marker difference. Regular connecting lines represent two marker differences. MLVA types connected by a same background could be considered a clonal complex. Asterisks in the MLVA types indicate the presence of tetracycline-and/or ofloxacin-resistant isolates.

The genetic relationships of the 210 isolates were deduced by construction of an MST (Figure 1). This population model highlighted one major clonal complex, composed of 207 isolates belonged to 38 MLVA types. A second clonal complex could be defined for one urogenital isolate (Mh-3560) collected in 2003 and yielding the MLVA type 24. A third clonal complex was represented by two respiratory isolates (Mh-2327, Mh-2477) collected six months apart in 1996–1997 from the same patient and were of the MLVA type 33. Interestingly, the two VNTRs that were not used for the MLVA assay (due to lack of discrimination) presented size variation only with these two isolates and harboured identical TR numbers between both isolates. The MST population modelling indicated the genetic diversity among the isolates tested. Unfortunately, there was no obvious link between the MLVA type and the isolate year of collection, the patient’s age or sex, the anatomical site of collection, or the antibiotic resistance. Indeed, the tetracycline or ofloxacin-resistant isolates were dispersed into 25 MLVA types (Figure 1). It should be noted that no significant difference in the repartition of MLVA types was identified between *M. hominis* cervical isolates present in ≥ 10^4^ CCU /ml in patients without BV and in patients with BV (Additional file 1: Table S1).

## Discussion

In this study, we present an MLVA-based molecular typing system for the discrimination of *M. hominis* isolates. We used this method on a group of 210 temporally separated isolates from French patients and obtained from a variety of urogenital and extragenital clinical circumstances. This effort represents the most comprehensive *M. hominis* molecular typing study because, until now, other studies were realised only on urogenital isolates and few isolates were tested [7-10].

MLVA typing of *M. hominis* is important both individually and epidemiologically. At the level of an individual patient, this approach allows discrimination between relapse or persistence and new infections. In the first case, the MLVA type remains identical. In the case of a reinfection, the MLVA type is likely to be different. Our MLVA scheme was used to study the course of infection in seven patients. In six of these patients, sequential isolates belonged to a consistent MLVA type in each case studied, suggesting in a persistent or relapse infection. Interestingly, the two clinical isolates Mh-2377 and Mh-2477 harboured the unique MLVA type 33 whereas previous PFGE analysis showed different migrations patterns when evaluated according to the interpretation guidelines of Tenover *et al*., and the total genome sizes of the two strains, deduced from the addition of the generated fragment lengths, were nearly identical [24]. These respiratory isolates were collected six months apart from a man with a chronic obstructive pulmonary disease who was treated several times with ciprofloxacin. As the *M. hominis* isolates were both resistant to fluoroquinolones, it would seem logical that the two isolates were identical, as shown by MLVA typing. The observed differences in PFGE patterns may be due to restriction sites located in variable regions or to recombination. Indeed, results from previous analysis indicated that a high levels of intragenic and intergenic recombination occurred in *M. hominis*, and these recombination levels are presumably important for the adaptation potential of this species [11,14].

Analysis of our results suggests a new infection in a female patient, as the two sequential cervical isolates were of different MLVA types. A previous study investigated cervical isolates of *M. hominis* obtained before and after treatment by RAPD. In two of nine cases studied, the profile of amplification did not change, whereas in the rest of cases, RAPD patterns were different, suggesting that the patients were reinfected [10].

We also performed molecular investigations of *M. hominis* isolates from two mother-neonate pairs. In each case studied, an identical MLVA type was found, confirming mother-to-child transmission. Our results are in agreement with those of Jensen et *al*. who reported that *M. hominis* isolates obtained from the cervices of pregnant women and from the ears or pharynges of their new-born infants yielded the same genomic profile by PFGE [7]. Similar results were obtained by Grattard et *al.*, who showed that strains isolated within a mother-neonate pair exhibited an identical pattern by AP-PCR [25].

At the population level, MLVA typing assesses the genetic diversity of *M. hominis* strains. In this study, we described 40 MLVA types, revealing a genetic heterogeneity among this species. This finding is in agreement with the data obtained by studies using other molecular typing methods. Using RFLP, Busch et *al*. found a high heterogeneity among 20 isolates obtained from colonised women and women with various urogenital infections [8]. The results from an AFLP analysis of five randomly chosen clinical isolates also provided further evidence of high-level intraspecies variability in this organism [9]. Soroka et *al*. reported genetic heterogeneity of genomes of *M. hominis* isolates using RAPD, and their results were confirmed by PFGE [10]. In comparison to the molecular typing methods that the other studies have used, MLVA is a reproducible and fast technique that does not require a sequencing step and can be standardised, facilitating large-scale molecular epidemiological investigations. The capillary electrophoresis on a genetic analyser enables high throughput analysis and allows easier interpretation of results (in contrast to agarose gel electrophoresis), particularly for VNTRs with a small number of repeat units.

In *M. hominis*, a high level of resistance to tetracyclines has been associated with the presence of the *tet*(M) determinant, the sole tetracycline resistance mechanism acquired by clinical isolates of human mycoplasmas [26]. It has been reported that in Bordeaux, France, the percentage of *M. hominis* isolates resistant to tetracyclines increased significantly, from 2.8% to 18.75%, between 1999 and 2002 [27]. In our study, the 68 urogenital *M. hominis* isolates resistant to tetracyclines were not related and clustered into 25 MLVA types, suggesting the absence of a link between tetracycline resistance and this typing method. Our results are in agreement with those of Mardassi et *al.*, who recently showed that resistance rates to tetracyclines were 25% among Tunisian *M. hominis* isolates and that molecular typing based on the nucleotide sequences of P120’ gene fragments indicated that these isolates were not clonal [28].

## Conclusions

This study represents the first attempt to perform molecular typing of a consequential number of *M. hominis* clinical isolates using the MLVA method. The VNTR analysis provides a rapid, simple molecular typing technique that has demonstrated its usefulness at the individual level. This new typing tool revealed a high genetic heterogeneity among *M. hominis* isolates, and seems too discriminatory to be used for epidemiological studies at a population level.

## Competing interests

The authors have declared that no competing interests exist.

## Authors’ contributions

CF and OP carried out the molecular studies, participated in the MST analysis and drafted the manuscript. HR participated in the molecular studies. CB conceived the design of the study, participated in its design and coordination and drafted the manuscript. All of the authors read and approved the final manuscript.

## Supplementary Material

Additional file 1: Table S1 Characteristics of the 210 *M. hominis* isolates used in this study. Click here for file

Additional file 2: Figure S1 Alignment of the sequences of the five targeted genomic regions of the 12 *M. hominis* strains used for the selection of the VNTRs. Click here for file

Additional file 3: Table S2 Oligonucleotide primers used for MLVA. Click here for file
