# Supplementary material for: Diversity of Mycoplasma hominis clinical isolates from Bordeaux, France, as assessed by multiple-locus variable-number tandem repeat analysis
Source: BMC Microbiol. 2013 May 28;13:120. doi: 10.1186/1471-2180-13-120 (PMC3694145; doi:10.1186/1471-2180-13-120)
Supplement: Additional file 1: Table S1 — Characteristics of the 210 M. hominis isolates used in this study. [file 1471-2180-13-120-S1.pdf]

Table S1. Characteristics of the 210 *M. hominis* isolates used in this study.

Isolate[a]	Sex	Origin		Year of isolation	MLVA type
		French city	Source		
Reference strains					
**PG21**[b]					36
**H34**					10
**M132**					10
Urogenital clinical isolates					
Commensal isolates					
Mh-5006	M	Bordeaux	urine	2009	5
Mh-5007	M	Bordeaux	urine	2009	8
Mh-5008	F	Bordeaux	cervix	2009	7
Mh-5011	M	Bordeaux	sperm	2009	12
Mh-5012	M	Bordeaux	sperm	2009	12
Mh-5015	M	Bordeaux	sperm	2009	17
Mh-5019	M	Bordeaux	sperm	2009	17
Mh-5021	M	Bordeaux	sperm	2009	7
Mh-5026	M	Bordeaux	sperm	2009	10
Mh-5027	M	Bordeaux	sperm	2009	4
Mh-5033	M	Bordeaux	urine	2009	8
Mh-5038	M	Bordeaux	urine	2009	8
Mh-5041	M	Bordeaux	sperm	2009	12
Mh-5044[S1]	M	Bordeaux	sperm	2009	10
Mh-5047 [S1]	M	Bordeaux	sperm	2009	10
Mh-5049	M	Bordeaux	urethra	2009	31
Mh-5061	M	Bordeaux	urethra	2009	8
Mh-5062	F	Bordeaux	cervix	2009	8
Mh-5070	M	Bordeaux	sperm	2009	9
Mh-5098	M	Bordeaux	sperm	2009	4

Isolates present in significant quantity in the sample (≥ 10^4 CCU/ml) without bacterial vaginosis					
Mh-2426	F	Bordeaux	cervix	1997	9
Mh-2987	F	Bordeaux	cervix	2000	10
Mh-3065	F	Bordeaux	cervix	2000	5
Mh-3068	F	Bordeaux	cervix	2000	8
Mh-3070	F	Bordeaux	cervix	2000	8
Mh-3076	F	Bordeaux	cervix	2000	29
Mh-3102	F	Bordeaux	cervix	2000	8
Mh-3273	F	Bordeaux	cervix	2001	8
Mh-3311	F	Bordeaux	cervix	2002	13
Mh-3316	F	Bordeaux	cervix	2002	9
Mh-3332	F	Bordeaux	cervix	2002	10
Mh-3348	F	Bordeaux	cervix	2002	7
Mh-3363	F	Bordeaux	cervix	2002	3
Mh-3404	F	Bordeaux	cervix	2002	12
Mh-3417	F	Bordeaux	cervix	2002	5
Mh-3425	F	Bordeaux	cervix	2002	4
Mh-3430	F	Bordeaux	cervix	2003	3
Mh-3431	F	Bordeaux	cervix	2003	8
Mh-3444	F	Bordeaux	cervix	2003	10
Mh-3482	F	Bordeaux	cervix	2003	5
Mh-3500	F	Bordeaux	cervix	2003	20
Mh-3512	F	Bordeaux	cervix	2003	27
Mh-3525	F	Bordeaux	cervix	2003	4
Mh-3534	F	Bordeaux	cervix	2003	39
Mh-3560	F	Bordeaux	cervix	2003	24
Mh-3567	F	Bordeaux	cervix	2003	10
Mh-3601	F	Bordeaux	cervix	2003	21
Mh-3623	F	Bordeaux	cervix	2004	8
Mh-3668	F	Bordeaux	cervix	2004	5

Mh-3716	F	Bordeaux	cervix	2004	13
Mh-3757	F	Bordeaux	cervix	2004	20
Mh-3853	F	Bordeaux	cervix	2005	7
Mh-3878	F	Bordeaux	cervix	2005	10
Mh-3890	F	Bordeaux	cervix	2005	18
Mh-3902	F	Bordeaux	cervix	2005	8
Mh-3956	F	Bordeaux	cervix	2005	5
Mh-3987	F	Bordeaux	cervix	2005	10
Mh-3998	F	Bordeaux	cervix	2005	8
Mh-4073	F	Bordeaux	cervix	2006	1
Mh-4089	F	Bordeaux	cervix	2006	40
Mh-4096	F	Bordeaux	cervix	2006	8
Mh-4206	F	Bordeaux	cervix	2006	27
Mh-4218	F	Bordeaux	cervix	2006	27
**Mh-4244**	F	Bordeaux	cervix	2006	4
Mh-4299	F	Bordeaux	cervix	2006	10
Mh-4362	F	Bordeaux	cervix	2006	21
Mh-4426	F	Bordeaux	cervix	2007	28
Mh-4457	F	Bordeaux	cervix	2007	8
Mh-4494	F	Bordeaux	cervix	2007	12
Mh-4563	F	Bordeaux	cervix	2007	29
Mh-4598	F	Bordeaux	cervix	2007	8
Mh-4634	F	Bordeaux	cervix	2007	4
Mh-4642[S3]	F	Bordeaux	cervix	2007	2
Mh-4652	F	Bordeaux	cervix	2007	10
Mh-4659	F	Bordeaux	cervix	2007	29
Mh-4697	F	Bordeaux	cervix	2007	5
**Mh-4704**	F	Bordeaux	cervix	2008	12
Mh-4705	F	Bordeaux	cervix	2007	20
Mh-4724[S3]	F	Bordeaux	cervix	2007	8

Mh-4732	F	Bordeaux	cervix	2007	8
Mh-4741	F	Bordeaux	cervix	2008	7
Mh-4744[S4]	F	Bordeaux	cervix	2008	10
Mh-4750	F	Bordeaux	cervix	2008	8
Mh-4754	F	Bordeaux	cervix	2008	4
Mh-4759	F	Bordeaux	cervix	2008	11
Mh-4768	F	Bordeaux	cervix	2008	5
Mh-4769	F	Bordeaux	cervix	2008	5
Mh-4777	F	Bordeaux	cervix	2008	20
Mh-4786	F	Bordeaux	cervix	2008	13
Mh-4788 [S4]	F	Bordeaux	cervix	2008	10
Mh-4809	F	Bordeaux	cervix	2008	15
Mh-4813	F	Bordeaux	cervix	2008	3
Mh-4819	F	Bordeaux	cervix	2008	8
Mh-4838	F	Bordeaux	cervix	2008	22
Mh-4842	F	Bordeaux	cervix	2008	5
Mh-4858	F	Bordeaux	cervix	2008	2
Mh-4866	F	Bordeaux	cervix	2008	5
Mh-4867	F	Bordeaux	cervix	2008	8
Mh-4869	F	Bordeaux	cervix	2008	38
Mh-4871	F	Bordeaux	cervix	2008	12
Mh-4873	F	Bordeaux	cervix	2008	5
Mh-4874	F	Bordeaux	cervix	2008	5
Mh-4877	F	Bordeaux	cervix	2008	10
Mh-4901	F	Bordeaux	cervix	2008	30
Mh-4916	F	Bordeaux	cervix	2008	7
Mh-4918	F	Bordeaux	cervix	2008	5
Mh-4922	F	Bordeaux	cervix	2008	5
**Mh-4927**	F	Bordeaux	cervix	2008	8
Mh-4946	F	Bordeaux	cervix	2008	32

Mh-4962	F	Bordeaux	cervix	2008	8
Mh-4971 [S5]	F	Bordeaux	cervix	2009	12
Mh-5002 [S5]	F	Bordeaux	cervix	2009	12
Mh-5060	F	Bordeaux	cervix	2009	8
Mh-5151 [S2]	F	Bordeaux	cervix	2009	5
Isolates present in quantity ≥ 10^4 CCU/ml and *associated with bacterial vaginosis*					
Mh-2993	F	Bordeaux	cervix	2000	10
Mh-2994	F	Bordeaux	cervix	2000	35
Mh-3017	F	Bordeaux	cervix	2000	7
Mh-3066	F	Bordeaux	cervix	2000	5
Mh-3110	F	Bordeaux	cervix	2001	10
Mh-3138	F	Bordeaux	cervix	2001	40
Mh-3146	F	Bordeaux	cervix	2001	5
Mh-3392	F	Bordeaux	cervix	2002	39
Mh-3614	F	Bordeaux	cervix	2004	20
Mh-3759	F	Bordeaux	cervix	2004	19
Mh-3924	F	Bordeaux	cervix	2005	7
Mh-4156	F	Bordeaux	cervix	2006	5
Mh-4403	F	Bordeaux	cervix	2006	10
Mh-4407	F	Bordeaux	cervix	2007	9
Mh-4454	F	Bordeaux	cervix	2007	5
Mh-4585	F	Bordeaux	cervix	2007	10
Mh-4635	F	Bordeaux	cervix	2007	23
Mh-4696	F	Bordeaux	cervix	2007	8
Mh-4734	F	Bordeaux	cervix	2008	8
Mh-4749	F	Bordeaux	cervix	2008	3
Mh-4763	F	Bordeaux	cervix	2008	4
Mh-4767	F	Bordeaux	cervix	2008	8
Mh-4775	F	Bordeaux	cervix	2008	18
Mh-4818	F	Bordeaux	cervix	2008	8

Mh-4904	F	Bordeaux	cervix	2008	8
Mh-4923	F	Bordeaux	cervix	2008	15
Mh-4963	F	Bordeaux	cervix	2008	25
Mh-5105	F	Bordeaux	cervix	2009	13
Isolates obtained from pregnant women with threatened preterm delivery					
Mh-5058	F	Bordeaux	placenta	2009	10
Mh-5059	F	Bordeaux	cervix	2009	10
Mh-5063	F	Bordeaux	cervix	2009	12
Mh-5072	F	Bordeaux	cervix	2009	11
Mh-5079	F	Bordeaux	cervix	2009	8
Mh-5086	F	Bordeaux	cervix	2009	4
Mh-5094	F	Bordeaux	cervix	2009	20
Mh-5096	F	Bordeaux	cervix	2009	15
Mh-5100	F	Bordeaux	cervix	2009	7
Mh-5134	F	Bordeaux	cervix	2009	7
Mh-5143	F	Bordeaux	gastric aspirate	2009	20
Mh-5149[S2]	F	Bordeaux	placenta	2009	5
Mh-5154	F	Bordeaux	cervix	2009	34
Mh-5160	F	Bordeaux	cervix	2009	34
Isolates from the upper genital tract					
Mh-2369	F	Bordeaux	endometrial biopsy	1997	3
Mh-3015	F	Bordeaux	douglas fluid	2000	8
Mh-3242	F	Bordeaux	douglas fluid	2001	16
Mh-3480	F	Bordeaux	amniotic fluid	2003	4
Mh-3561	F	Bordeaux	hydrosalpinx	2003	5
**Mh-3631**	F	Bordeaux	amniotic fluid	2004	4
Mh-4016	F	Lannion	endometrial biopsy	2005	8
Mh-4235	F	Bordeaux	peritoneal fluid	2006	10
Mh-4752	F	Bordeaux	peritoneal fluid	2008	4
Mh-4970	F	Bordeaux	peritoneal fluid	2008	39

**Mh-4829**	F	Bordeaux	peritoneal fluid	2008	3
Extragenital isolates					
Mh-129	F	Bordeaux	blood culture	1990	37
Mh-331	M	Bordeaux	joint fluid	1991	15
Mh-338	F	Bordeaux	joint fluid	1991	15
Mh-785	M	Nantes	renal abscess after transplantation	1993	3
Mh-1323	M	Pontoise	mediastinitis	1995	26
**Mh-1562**	F	Bordeaux	joint fluid	1995	3
Mh-2052	M	Bordeaux	pleural fluid	1996	8
Mh-2327 [S6]	M	Paris	bronchial aspirate	1996	33
Mh-2382	F	Bordeaux	joint fluid	1997	15
Mh-2446	M	Paris	wound facial surgery	1997	9
**Mh-2477** [S6]	M	Paris	bronchial aspirate	1997	33
Mh-2478	M	Paris	mediastinitis	1997	15
Mh-2513	M	Paris	renal abscess after transplantation	1997	5
**Mh-2537**[C]	M	Marseille	abdominal wall abscess	1997	3
Mh-2539[C]	M	Marseille	broncho-alveolar fluid	1997	3
Mh-2668	F	Saint-Brieuc	broncho-alveolar fluid	1998	22
Mh-2674	M	Paris	mediastinitis	1998	10
Mh-2712	M	Clermont-Ferrand	renal abscess after transplantation	1998	22
Mh-2769	F	Bordeaux	joint fluid	1999	14
Mh-2781	F	Lannion	blood culture	1999	8
Mh-2789	M	Toulouse	broncho-alveolar fluid	1999	10
Mh-2814	M	Paris	mediastinitis	1999	5
Mh-2899 [S7]	F	Paris	broncho-alveolar fluid	2000	10
Mh-2900 [S7]	F	Paris	broncho-alveolar fluid	2000	10
Mh-2997	M	Paris	sternal wound	2000	9
Mh-3210	F	Lannion	abdominal wall abscess	2001	9
Mh-3265	M	Bordeaux	sternal wound	2001	10
Mh-3299	F	Bordeaux	joint fluid	2002	36

Mh-3339	M	Paris	cerebral hematoma	2002	8
Mh-3364	M	Paris	joint fluid	2002	6
Mh-3665	M	Orléans	broncho-alveolar fluid	2004	10
Mh-4285	M	Paris	renal abscess after transplantation	2006	3
Mh-4475	M	Paris	renal abscess after transplantation	2007	3
**Mh-4796**	M	Paris	renal abscess after transplantation	2008	17
Isolates from mothers and their neonate					
Mh-33	F	Bordeaux	blood culture (mother 1)	1989	8
Mh-35	F	Bordeaux	blood culture (neonate 1)	1989	8
Mh-352	F	Bordeaux	blood culture (mother 2)	1991	7
Mh-358	F	Bordeaux	cervix (mother 2)	1991	7
Mh-353	F	Bordeaux	blood culture (neonate 2)	1991	7
Mh-360	F	Bordeaux	throat (neonate 2)	1991	7

[a] Sequential and concomitant isolates are indicated by the letter S (S1 to S7) or C, respectively.

[b] Isolates in bold were used for the selection of the VNTR markers.
