# Supplementary material for: Diversity of Mycoplasma hominis clinical isolates from Bordeaux, France, as assessed by multiple-locus variable-number tandem repeat analysis
Source: BMC Microbiol. 2013 May 28;13:120. doi: 10.1186/1471-2180-13-120 (PMC3694145; doi:10.1186/1471-2180-13-120)
Supplement: Additional file 2: Figure S1 — Alignment of the sequences of the five targeted genomic regions of the 12 M. hominis strains used for the selection of the VNTRs. [file 1471-2180-13-120-S2.pdf]

Mho-50

```
      1                                                                                                  100
PG21  AAACAACAAATGATAGTCAAAATTCTACAACCACAGGTGAAGATTCGACTGGACAATCTCAAGATTCTACAACCACAGGTGAAGATTCGACTGGACAATC
H34   AAACAACAAATGATAGTCAAGATTCTACGACCACAGGTGAAGATTCGACCGGGCAATC------------------------------------------
M132  AAACAACAAATGATAGTCAAGATTCTACGACCACAGGTGAAGATTCGACCGGGCAATC------------------------------------------
4244  AAACAACAAATGATAGTCAAGATTCTACGACCACAGGTGAAGATTCGACCGGACAATC------------------------------------------
4704  AAACAACAAATGATAGTCAAGATTCTACGACCACAGGTGAAGATTCGACCGGACAATC------------------------------------------
                                         1                                         2
4927  AAACAACAAATGATAGTCAAGATTCTACGACCACAGGTGAAGATTCGACCGGACAATC------------------------------------------
3631  AAACAACAAATGATAGTCAAGATTCTACGACCACAGGTGAAGATTCGACCGGACAATC------------------------------------------
4829  AAACAACAAATGATAGTCAAGATTCTACGACCACAGGTGAAGATTCGAGTGGGCAATC------------------------------------------
1562  AAACAACAAATGATAGTCAAGATTCTACGACCACAGGTGAAGATTCGACCGGACAATC------------------------------------------
2477  AAACAACAAATGATAGTCAAAATTCTACAACCACAGGTGAAGATTCGACTGGACAATCTCAAGATTCTACGACCACAGGTGAAGATTCGACTGGACAATC
2537  AAACAACAAATGATAGTCAAAATTCTACAACCACAGGTGAAGATTCGAGTGGGCAATC------------------------------------------
4796  AAACAACAAATGATAGTCAAAATTCTACAACCACAGGTGAAGATTCGACTGGACAATC------------------------------------------

      101                                                                                                200
PG21  TCAAGATTCTACAACCACAGGTGAAGATTCGAGTGGACAATCTCAAGATTCTACAACCACAGGTGAAGATTCGAGTGGACAATCGCAATCTGGCGACATT
H34   ------------------------------------------------------------------------------------TCAATCTGGCGACATT
M132  ------------------------------------------------------------------------------------TCAATCTGGCGACATT
4244  ------------------------------------------------------------------------------------TCAATCTGGCGACATT
4704  ------------------------------------------------------------------------------------TCAATCTGGCGACATT
                          3                                         4
4927  ------------------------------------------------------------------------------------TCAATCTGGCGACATT
3631  ------------------------------------------------------------------------------------TCAATCTGGCGACATT
4829  ------------------------------------------------------------------------------------TCAATCTGGCGACATT
1562  ------------------------------------------------------------------------------------TCAATCTGGCGACATT
2477  TCAAGATTCTACGACCACAGGTGAAGATTCGACCGGACAATCTCAAGATTCTACGACCACAGGTGAAGATTCGACCGGACAATCTCAATCTGGCGACATT
2537  ------------------------------------------------------------------------------------TCAATCTGGCGACATT
4796  ------------------------------------------------------------------------------------GCAATCTGGCGACATT
```

Mho-52

```
       1                                                                                              96
PG21   GACATAAATTCGTTAGATGATGAAGTTGCTCATGCGGCAGCTAAGGATGATGATAATGATGACGATGAT---GCAGAAGAGGAAGAAAAAGATTTT
H34    GACATAAATTCGTTAGATGATGAAGTTGCTCATGCAACAACTAAAGATGATGATAACGATGACGATGAT---GCAGAAGAGGAAGAAAAAGATTTT
M132   GACATAAATTCGCTAGATGATGAAGTTGCTCACGCAACAACTAAAGATGATGATAATGATGACGATGAC---GCAGAAGAGGAAGAAAAAGATTTT
4244   GACATAAATTCGTTAGATGATGAAGTTGCTCACGCAACAACTAAAGATGATGATAATGATGACGATGAC---GCAGAAGAGGAAGAAAAAGATTTT
4704   GACATAAATTCGCTAGATGACGAAGTTGCTCACGCAACAACTAAAGATGATGATAATGATGACGATGAC---GCAGAAGAGGAAGAAAAAGATTTT
4927   GACATAAATTCGCTAGATGATGAAGTTGCTCACGCAACAACTAAAGATGATGATAATGATGACGATGAC---GCAGAAGAGGAAGAAAAAGATTTT
                                                     1  2  3  4  5  6  7  8  9
4829   GACATAAATTCGCTAGATGATGAAGTTGCTCACGCAACAACTAAAGATGATGATAATGATGACGATGAC---GCAGAAGAGGAAGAAAAAGATTTT
3631   GACATAAATTCGCTAGATGATGAAGTTGCTCACGCAACAACTAAAGATGATGATAATGATGACGATGAC---GCAGAAGAGGAAGAAAAAGATTTT
1562   GACATAAATTCGCTAGATGATGAAGTTGCTCACGCAACAACTAAAGATGATGATAATGATGACGATGAC---GCAGAAGAGGAAGAAAAAGATTTT
2477   GACATAAATTCGTTAGATGATGAAGTTGCTCACGCAACAACTAAAGATGATGATAATGATGACGATGAC---GCAGAAGAGGAAGAAAAAGATTTT
2537   GACATAAATTCGCTAGATGACGAAGTTGCTCACGCAACAACTAAAGATGATGATAATGATGACGATGAC---GCAGAAGAGGAAGAAAAAGATTTT
4796   GACATAAATTCGTTAGATGATGAAGTTGCTCATGCGGCAGCTAAAGATGATGATAACGATGATGATGATGATGCAGAAGAGGAAGAAAAAGATTTT
```

Mho-53

```
       1                                                                           77
PG21   GGCGATGGGCTAGAATCTTGATTTGATTTATTATTATTATTATTAAT---TTGATCTTGATTATTTTTATTTGTTGC
H34    GGCGATGGGCTAGAATCTTGATTTGATTTATTATTATTATTATTATT---TTGATCTTGATTATTTTTATTTGTTGC
M132   GGCGATGGGCTAGAATCTTGATTTGATTTATTATTATTATTATTATT---TTGATCTTGATTATTTTTATTTGTTGC
4244   GGCGATGGGCTAGAATCTTGATTTGATTTATTATTATTATT---------TTGATCTTGATTATTTTTATTTGTTGC
4704   GGCGATGGGCTAGAATCTTGATTTGATTTATTATTATTATTATTATTATTTTGATCTTGATTATTTTTATTTGTTGC
4927   GGCGATGGGCTAGAATCTTGATTTGATTTATTATTATTATTATT------TTGATCTTGATTATTTTTATTTGTTGC
                                     1  2  3  4  5  6  7
3631   GGCGATGGGCTAGAATCTTGATTTGATTTATTATTATTATT---------TTGATCTTGATTATTTTCATTTGTTGC
4829   GGCGATGGGCTAGAATCTTGATTTGATTTATTATTATT------------TTGATCTTGATTATTTTCGTTTGTTGC
1562   GGCGATGGGCTAGAATCTTGATTTGATTTATTATTATT------------TTGATCTTGATTATTTTCGTTTGTTGC
2477   GGCGATGGGCTAGAATCTTGATTTGATTTATTATTATT------------TTGATCTTGATTATTTTTATTTGTTGC
2537   GGCGATGGGCTAGAATCTTGATTTGATTTATTATTATT------------TTGATCTTGATTATTTTTATTTGTTGC
4796   GGCGATGGGCTAGAATCTTGATTTGATTTATTATTATTATT---------TTGATCTTGATTATTTTTATTTGTTGC
```

Mho-114

```
     1                                                                                              100
PG21 TGTAATAATCTGAAGTTTTAACATCAGGAAATATTTCTCTTCAATTAATCTCTGTTTTTATAATTGGCTTTGGCTTTGTTTCTGGTTTAAGCTTTTTATC
H34  TGTAATAATCTGAAGTTTTAACATCAGGAAATATTTCTCTTCAATTAATCTCTGTTTTTATATTCAGCCTTGGCTTTGCTTCTGGTTTATGCTCTTTATC
M132 TGTAATAATTTGAAGTTTTAACATCAGGAAATATTTCTCTTCAGTTAATCTCTGTTTTTATATTTGGCCTTGGCTTTGCTTCTGGTTTATGCTCTTTATC
4244 TGTAATAATCTGAAGTTTTAACATCTGGAAATATTTCTCTTCAATTAATCTCTGTTTTTATAATTGGCT------TTGTTTCTGGTTTAAGCTTTTTATC
4704 TGTAATAATCTGAAGTTTTAACATCAGGAAATATTTCTCTTCAGTTAATCTCTGTTTTTATATTCGGCCTTGGCTTTGCTTCTGGTTTATGCTCTTTATC
4927 TGTAATAATCTGAAGTTTTAACATCAGGAAATATTTCTCTTCAATTAATCTCTGTTTTTATAATTGGCTTTGGCTTTGTTTCTGGTTTAAGCTCTTTATC
3631 TGTAATAATCTGAAGTTTTAACATCTGGAAATATTTCTCTTCAATTAATCTCTGTTTTTATAATTGGCT------TTGTTTCTGGTTTAAGCTTTTTATC
4829 TGTAATAATCTGAAGTTTTAACATCAGGAAATATTTCTCTTCAATTAATCTCTGTTTTTATATTCGGCCTTGGCTTTGCTTCTGGTTTATGCTCTTTATC
1562 TGTAATAATCTGAAGTTTTAACATCAGGAAATATTTCTCTTCAATTAATCTCTGTTTTTATATTTGGCCTTGGCTTTGCTTCTGGTTTAAGCTTTTTATC
2477 TGTAATAATCTGAAGTTTTAACATCAGGAAATATTTCTCTTCAATTAATCTCTGTTTTTATAATTGGCTTTGGCTTTGTTTCTGGTTTAAGCTCTTTATC
2537 TGTAATAATCTGAAGTTTTAACATCAGGAAATATTTCTCTTCAGTTAATCTCTGTTTTTATATTCGGCCTTGGCTTTGCTTCTGGTTTATGCTCTTTATC
4796 TGTAATAATCTGAAGTTTTAACATCAGGAAATATTTCTCTTCAATTAATCTCTGTTTTTATAATTGGCTTTGGCTTTGTTTCTGGTTTAAGCTCTTTATC
```

Mho-116

```
     1                                                                                              100
PG21 TTTTCCAAAATCGAATTCGATTGGTTTTGGTTTTGAA---CCTCTAACATCTTTTTTAGCAATTACATATTTTCCAGTTTTAGGATCTTTTTCTAATTTA
H34  TTTTCCAAAATCGAATTCAATTGGTTTTGGTTTTGAA---CCTTTAACAGTTTTTTTAGCAATTACATATTTTTTAGTATTGGCATCTTGTTCTAATTTA
M132 TTTTCCAAAATCGAATTCGATTGGTTTTGGTTTTGAA---CCTTTAACAGTTTTTTTAGCAATTACATATTTTTTAGTATTGGTATCTTGTTCTAATTTA
4244 TTTTCCAAAATCGAATTCGATTGGTTTTGGTTTTGAA---CCTTTAACATCTTTTTTGGCAATTACATATTTTTTAGTATTGGTATCTTGTTCTAATTTA
4704 TTTTCCAAAATCGAATTCAATTGGTTTTGGTTTTGAA---CCTTTAACATCTTTTTTAGCAATTACATATTTTCCAGTTTTAGGATCTTTTTCTAATTTA
4927 TTTTCCAAAATCGAATTCAATTGGTTTTGGTTTTGAA---CCTTTAACATCTTTTTTAGCAATTACATATTTTCCAGTTTTAGGATCTTTTTCTAATTTA
3631 TTTTCCAAAATCGAATTCAATTGGTTTTGGTTTTGAA---CCTTTAACAGTTTTTTTAGCAATTACATATTTTTTAGTATTGGTATCTTGTTCTAATTTA
4829 TTTTCCAAAATCGAATTCGATTGGTTTTGGTTTTGAA---CCTTTAACAGTTTTTTTAGCAATTACATATTTTTTAGTATTGGTATCTTGTTCTAATTTA
1562 TTTTCCAAAATCGAATTCGATTGGTTTTGGTTTTGAA---CCTTTAACAGTTTTTTTAGCAATTACATATTTTTTAGTATTGGTATCTTGTTCTAATTTA
2477 TTTTCCAAAATCGAATTTAATTAGTTCTGGTGTTGAAGAACCTTTAGCATATCTTTCATCAATTACATATTTCCCGGTTTTAAGATCTTTTTCGAATTTA
2537 TTTTCCAAAATCGAATTCAATTGGTTTTGGTTTTGAA---CCTCTAACATCTTTGTTAGCAATTACATATTTTTTAGTATTGGCATCTTGTTCTAATTTA
4796 TTTTCCAAAATCGAATTCGATTAGTTTTGGTTTTGAA---CCTTTAACAGTTTTTTTAGCAATTACATATTTTTTAGTATTGGTATCTTGTTCTAATTTA
```

Figure S1. Alignment of the sequences of the five targeted genomic regions of the 12 *M. hominis* strains used for the selection of the VNTRs. For each VNTR, repeat copies are boxed and marked by the number corresponding to the number of the repeat marker. Numbers indicate on the left of the alignment designate *M. hominis* strain names.
