# Supplementary material for: Diversity of Mycoplasma hominis clinical isolates from Bordeaux, France, as assessed by multiple-locus variable-number tandem repeat analysis
Source: BMC Microbiol. 2013 May 28;13:120. doi: 10.1186/1471-2180-13-120 (PMC3694145; doi:10.1186/1471-2180-13-120)
Supplement: Additional file 3: Table S2 — Oligonucleotide primers used for MLVA. [file 1471-2180-13-120-S3.pdf]

Table S2. Oligonucleotide primers used for MLVA.

Primer name[a]	Dye sequence (5' → 3')
Mho-50-fwd	NED-GCAGCAGGATGCAAAACAA
Mho-50-rev	GGCCTAATTGACCTTTTGCTC
Mho-52-fwd	HEX-TTGAAAGCCTTTTTGCAGCTA
Mho-52-rev	TCGTCAAGATTTCTTCTTCCA
Mho-53-fwd	FAM-AGGCGATGGGCTAGAATCTT
Mho-53-rev	GCGTGAATTAGCGGCATTAT
Mho-114-fwd	TCAATAAATCCTTGGCCATCT
Mho-114-rev	FAM-CCAAGCGAATGATGACGATA
Mho-116-fwd	NED-TTTATCACGGCCAACAAACA
Mho-116-rev	TGCCTGCAATTTTAGCATCA

[a] fwd, forward primer; rev, reverse primer
